# The Effect of the Dietary Inclusion of Crude Glycerin in Pre-Starter and Starter Diets for Piglets

**DOI:** 10.3390/ani11051249

**Published:** 2021-04-26

**Authors:** Juan Orengo, Josefa Madrid, Juan Luis Aragón, Silvia Martínez-Miró, Miguel J. López, Fuensanta Hernández

**Affiliations:** 1 Department of Animal Production, Faculty of Veterinary Science, Regional Campus of International Excellence “Mare Nostrum”, University of Murcia, 30100 Murcia, Spain; alimen@um.es (J.M.); silviamm@um.es (S.M.-M.); mjlopeza@um.es (M.J.L.); 2 Mupesa Pecuaria, S.L., Polígono Ind. Las Salinas, Av. Europa, 29, Alhama de Murcia, 30849 Murcia, Spain; juanluis.aragon@mupesa.net

**Keywords:** crude glycerin, growth performance, metabolic hormones, nutrient digestibility, piglets

## Abstract

**Simple Summary:**

The surplus of crude glycerin and the rising cost of feedstuffs have encouraged the nutritional valorization of this by-product as an interesting alternative ingredient in pig feed. We tested the addition of 2.5 and 5% glycerin to pelleted piglet diets to assess the effects on growth performance and digestibility of weaned piglets and to determine the serum concentrations of hormones related to energy metabolism and feed intake. Crude glycerin was included as a replacement for sheep sweet whey and wheat in pre-starter and starter diets, respectively. Growth data and fecal samples were collected at the end of each feeding phase. At the end of the study, blood samples were taken to analyze insulin and ghrelin concentrations. Over the whole period, our results showed that the average daily gain and the feed conversion ratio were not affected negatively by the dietary glycerin inclusion. There were also no differences between dietary treatments in terms of blood metabolites measured while the digestibility coefficients of dry and organic matter improved as glycerin increased. Therefore, crude glycerin could be used as an energy source to replace sweet whey and wheat and be added to pre-starter and starter diets.

**Abstract:**

The aim of this study was to evaluate the effect of the inclusion of crude glycerin in post-weaning diets for piglets on growth performance and digestibility. The study was carried out with a total of 360 piglets over a 39 day period. Animals were blocked by body weight (7.7 ± 0.86 kg) and allotted randomly to one of three dietary treatments containing 0, 2.5 or 5% glycerin (G0, G2.5 and G5, respectively). Considering the whole period, glycerin did not affect the average daily gain. However, the average daily feed intake (ADFI) and the feed conversion ratio (FCR) tended to decrease or decreased linearly as the amount of glycerin increased, respectively (*p* = 0.060 and *p* = 0.039). The apparent total tract digestibility (ATTD) of dry and organic matter (DM, OM) increased linearly with increasing glycerin in both periods (*p* ≤ 0.05). At the end of the study, there were no differences between treatments for any of the hormones measured. In conclusion, the FCR and digestibility of DM and OM were improved although the ADFI tended to be lower when glycerin was included at 5%. Consequently, crude glycerin could be used as an alternative ingredient to partially replace sweet whey and wheat in post-weaning diets.

## 1. Introduction

Crude glycerin is the main by-product of the biodiesel industry, concerning about 10% of the biodiesel produced [1]. Biodiesel production has grown steadily over the past 20 years due to energy and environmental policies searching for alternative energy resources and in order to face the possible crisis of fossil fuel availability. The global production of biodiesel increased 13% in 2019 compared with 2018 to 47.4 billion liters [2]. The surplus of crude glycerin and the increase in feed ingredient prices have led to its evaluation as animal feed both in ruminant and non-ruminant species. In fact, a few studies have shown that crude glycerin is an interesting alternative as a feed ingredient for swine mainly in growing-finishing pigs and at a rate inclusion from 5 to 10% [3,4] or even higher [5]. Along these lines, the search for markets for crude glycerin also addresses the environmental problems associated with the generation of surplus [6] and where the reutilization and valorization of industrial byproducts are a major challenge toward sustainable development and a circular economy [7].

Several authors have also focused on the addition of glycerin to piglet diets as a partial replacement for cereals, mainly corn [3,8] or wheat [9]. In this sense, Lammers et al. [3] or Groesbeck et al. [8] showed that pigs could be fed corn-soybean meal-based diets including crude glycerin up to 6 or 10% without reducing pig performance while Zijlstra et al. [9] concluded that crude glycerin could be included up to 8% in weanling pig diets as replacement for wheat without reducing the growth and feed efficiency. However, few studies have used glycerin as a substitute for milk by-products and lactose-rich ingredients in newly weaned piglets [10,11,12]. Furthermore, Shields et al. [10] found different effects of replacing lactose with crude glycerin on post-weaning growth performance when comparing two experiments using the same source of glycerin and the same genetic line of piglets. On the other hand, glycerol did not appear to be a lactose replacement source for the first 21 post-weaning days [13]. 

It is well known that lactose allows a smoother transition from milk to solid feed, increasing the palatability of the diet and the growth performance of piglets during the post-weaning period [14,15,16]. These effects are also related to the limited capacity of the gastrointestinal tract to digest and effectively use starch because of a low level of carbohydrases other than lactase. Thus, it is widely accepted that piglets benefit from readily digestible carbohydrates until their digestive system and enzymatic activity are fully capable of hydrolyzing starch. In addition, the role of lactose as a potential prebiotic through the selective stimulation of the growth and/or activity of specific members of the gut microbiota and other possible benefits should be considered [17]. However, sustainable pig production requires that economics also be taken into account, as lactose and lactose-rich ingredients are expensive [17] and a potential alternative such as glycerin would be useful when prices are high.

Sweet whey or dried whey is commonly used as an excellent source of lactose (70%) in weanling diets. It is a byproduct resulting from the manufacture of hard cheeses and more common than acid whey (also known as sour whey) in Spain [18]. Similar to sweet whey, crude glycerin has a sweet taste, which could increase the palatability of feed [19] and is highly digestible in pigs [20]. This glycerin marketed for animal feed, included in the Catalogue of Feed Materials (13.8.1 or 13.8.2; EU Commission Reg. 575/2011), has a wide range of compositions: 70–93% glycerol, 8–20% moisture, 2–10% minerals (mainly rich in Na or K) and 100–750 mg/kg of methanol [18]. Moreover, it may become a cost-competitive alternative to partially substitute energy ingredients in piglet diets [21].

The aim of this study was to evaluate the effect of the dietary inclusion of crude glycerin by replacing partially sweet whey or wheat as energy sources in pre-starter and starter diets for piglets on growth performance and digestibility.

## 2. Materials and Methods

The experimental procedures were in compliance with the European Union regulations concerning the protection of animals used for experimental and other scientific purposes (EU Directive 2010/63/EU) and the study protocol was approved the by the Ethics Committee of the University of Murcia and the Authorities of the Region of Murcia (No. A-13170805).

### 2.1. Animals, Diets and Experimental Design

This experimental trial was performed on a commercial farm located in Purias-Lorca (Murcia, southeastern Spain). This study involved 360 crossbred piglets (½ Pietrain × ¼ Large White × ¼ Landrace). Piglets were weaned at 26 days of age with an initial body weight (BW) of 7.7 ± 0.86 kg. They were sorted and blocked by BW and balanced by sex within pens (50:50). Piglets were housed in 18 pens of 20 animals each and pens were assigned randomly to one of the three dietary treatments with six pens per treatment. Each pen (3 m × 1.5 m) had a self-feeder and two nipple drinkers to allow ad libitum access to food and water. 

Piglets were fed using a two-phase feeding program over a 39 day period. The pre-starter and starter phase comprised periods of 24 and 15 days, respectively. Experimental diets for both periods contained 0, 2.5 and 5% crude glycerin (G0, G2.5 and G5, respectively) and they were formulated to be isoenergetic and to have the same lysine:net energy ratio (Lys:NE) for each feeding period (pre-starter and starter) according to the recommendations of FEDNA (Fundación Española para el Desarrollo de la Nutrición Animal) [22]. Crude glycerin was included as a replacement for sheep sweet whey and wheat in pre-starter and starter diets, respectively. The energy value of crude glycerin considered in the feed formulation matrix was based on FEDNA [18]. The crude glycerin used in this study was from a biodiesel production facility (Abengoa Bioenergía San Roque, Cadiz, Spain), which used vegetables as feedstock. The composition of the diets is shown in Table 1. An indigestible marker (TiO_2_) was added at 0.5% to diets to calculate digestibility. All diets were presented in pelleted form.

### 2.2. Data Recording and Sampling

All animals were weighed individually at the beginning of the pre-starter and starter periods and at the end of the study. The average daily gain (ADG) was calculated on a pen basis from the individual BW of the pigs. Feed intake per pen was recorded by weighing feed added to the feeders and feed remaining at the end of each period to determine the average daily feed intake (ADFI) and the feed conversion ratio (FCR).

In order to calculate the apparent total tract digestibility (ATTD) coefficients of nutrients on each diet, fecal grab samples were collected directly from the anus of five randomly selected piglets in each pen at the end of each period (on day 24 and 39, respectively). The feces were pooled by pen and frozen at −20 °C for further analyses.

In addition, on day 39 of the study, 12 piglets were randomly selected for each dietary treatment (two piglets per pen) to analyze insulin and total and acylated ghrelin concentrations. Piglets within each pen were chosen according to their BW as being close to the average weight of the pen and feed was not withheld prior to sampling. Blood samples were collected via a jugular puncture into 4 mL vacuum tubes without additives (BD Vacutainer, Plymouth, UK). After centrifugation at 3500× *g* for 7 min at 4 °C, the serum was collected and stored at −80 °C until further analysis. 

### 2.3. Physical and Chemical Analyses

Two batches of each dietary treatment by feeding period (pre-starter and starter) were manufactured. The pellet durability was determined using a Holmen Pellet Tester (Holmen NHP100 Portable Pellet Tester; TekPro Ltd., Coleraine, UK). The pellet durability index (PDI) was measured from three 100 g subsamples of each batch. These subsamples were taken at regular intervals during the manufacturing process.

Chemical analyses were carried out according to the procedures of the AOAC (Association of Official Analytical Chemists) [23]. The dry matter (DM) content of the diets was determined by drying a sample in a convection oven at 105 °C for 8 h (method 934.01). The DM of feces was determined by drying at 60 °C in a forced-air drying oven until the sample reached a constant weight. Diet and fecal samples were ground through a 1 mm screen (ZM200, Retsch, Haan, Germany), and analyzed for crude protein (CP) by the Kjeldahl (984.13 A-D) method. The mineral content of the diets and feces was determined by dry ashing using a muffle furnace at 550 °C. Diet samples were also analyzed for crude fiber (962.09) and starch. The starch content was measured polarimetrically using the official analytical method described in BOE (Boletin Oficial del Estado) [24]. 

The titanium dioxide content of the diets and fecal samples was analyzed by the colorimetric method described by Myers et al. [25] and the coefficients of the ATTD were calculated from these data by using the formula ATTD (%) = (1 − ((nutrient in feces (g/kg DM)/nutrient in diet (g/kg DM)) × (TiO_2_ in diet (g/kg DM)/TiO_2_ in feces (g/kg DM)))) × 100.

Serum insulin and total and acylated ghrelin were quantified in duplicate using commercial radioimmunoassay kits (PI-12K, GHRT-89HK and GHRT-88HK, respectively; Linco Research, Saint Charles, MO, USA), which were previously validated for use in a porcine by Reynolds et al. [26]. The inter-assay CVs were less than 10% and the intra-assay CVs were less 11% for insulin and less than 6% for total and acylated ghrelin. The sensitivity for insulin, total ghrelin and acylated ghrelin was 1.6 µg/mL, 93 pg/mL and 10 pg/mL, respectively.

### 2.4. Statistical Analyses

Pellet durability, growth performance and digestibility data were analyzed by a linear model including only the fixed effect of the dietary treatment using the SPSS program (SPSS Inc., Chicago, IL, USA). For the analysis of the weight at the end of each feeding period, the model used also included the BW at the beginning of the period as a covariate. The pen was considered as the experimental unit. Serum data were logarithmically transformed (log 10) to meet the normality assumption; they were then analyzed using a mixed model to account for the effects of the dietary treatment, pen and residuals. The dietary treatment was considered to be a fixed effect whereas the pen and residuals were considered to be random effects. For serum data analyses, the animal was considered to be the experimental unit. All reported means were least square means and pairwise comparisons of means were performed using the least significant difference (LSD) test. Orthogonal contrasts were used to determine linear and quadratic effects of the dietary glycerin level. In addition, Pearson correlation coefficients were used to determine the relationship between concentrations of the serum metabolites. The results were considered significant at *p* ≤ 0.05 and a trend at *p* > 0.05 and *p* ≤ 0.10.

## 3. Results

**Figure 1:**
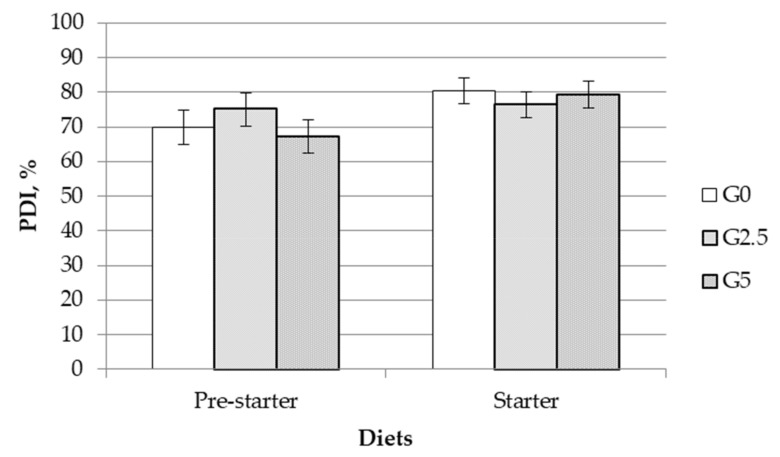
The effect of adding glycerin to feed on the Pellet Durability Index (PDI). Dietary treatments: 0, 2.5 and 5% crude glycerin included in G0, G2.5 and G5, respectively. Each bar represents the least square means of three feed samples. The error bars represent 95% confidence intervals of the mean. Non-significant effects were observed for the dietary treatment.

The effect of glycerin on pellet durability in post-weaning diets is represented in Figure 1. The inclusion of glycerin did not affect the PDI of either pre-starter or starter diets.

Table 2 shows the effect of including glycerin in the diet on growth performance. A quadratic response to the dietary glycerin addition was found (*p* = 0.026) for BW at the end of the pre-starter period. Piglets fed 2.5% glycerin showed a higher BW after 24 days of study than piglets fed 5% glycerin (15.60 vs. 14.77 kg) although there were no differences between glycerin-fed piglets and piglets fed the control diet. In the pre-starter period, glycerin-fed piglets had an increased ADG (quadratic, *p* ≤ 0.05) and a reduced ADFI (linear, *p* ≤ 0.05) compared with piglets fed the control diet. The higher ADG and ADFI were found in G2.5 and G0 diets, respectively, without differences between both treatments while piglets fed G5 had lower ADG and ADFI figures. For this period, the addition of crude glycerin decreased the FCR (linear and quadratic, *p* ≤ 0.05). The FCR for glycerin-fed piglets was 9% lower than for the piglets fed the G0 diet.

The dietary treatment had no effect on the final BW or ADG in the starter period. However, piglets fed the G2.5 diet had a higher feed intake but it was not different when compared with the control diet. This higher feed intake for piglets fed the G2.5 diet was reflected in its higher FCR (quadratic, *p* = 0.008). Nevertheless, the FCR was not different between piglets fed the G0 diet and those fed 5% glycerin.

Considering the whole period, the dietary treatment did not affect the ADG. However, the ADFI tended to decrease as the amount of glycerin increased (linear, *p* = 0.060) while a linear effect was found for the FCR (*p* = 0.039). Piglets fed the G5 diet showed a lower ADFI and FCR in comparison with piglets fed other dietary treatments.

The effects of glycerin inclusion on the ATTD of the pre-starter and starter diets are presented in Table 3. In general, crude glycerin affected the digestibility coefficient of all dietary fractions (DM, organic matter (OM), and CP) in both feeding periods: pre-starter and starter (*p* ≤ 0.05). The ATTD of DM and OM increased linearly (*p* ≤ 0.05) with increasing dietary glycerin in both periods. These digestibility coefficients were higher for diet G5 in comparison with those found for the other dietary treatments. For the ATTD of CP, a linear and a quadratic effect of the dietary inclusion of glycerin were found according to the period (pre-starter and starter period, respectively) (*p* ≤ 0.01). For both periods, the CP digestibility coefficients for diet G5 were numerically greater than 80% although this coefficient was not different to that found for the control diet in the starter period (80.3 vs. 78.1%).

The effect of glycerin inclusion on the serum concentration of ghrelin and insulin is presented in Table 4. At the end of the period studied, 39 days after weaning, there were no differences between the dietary treatments for any of the metabolites measured. Furthermore, a negative correlation was found between the total and acylated ghrelin (r = −0.686; *p* ≤ 0.05) while no significant correlations were found between both ghrelin forms and insulin (Table S1).

## 4. Discussion

Crude glycerin is a highly viscous liquid at room temperature, which has a difficult handling in the feed manufacturing process. A few researchers have shown that its pre-pelleting application improved production efficiency, reducing manufacturing costs [8,27]. Moreover, when the diet was offered in a pelleted form, the glycerin addition could affect the pellet quality. In this line, Groesbeck et al. [8] and Shields [28] reported that the PDI was improved with increasing levels of glycerin (up to 9 and 5%, respectively). However, in our study, there were no differences in the PDI when crude glycerin was added to piglet diets.

The use of crude glycerin in animal feeding is limited by the glycerol content, which is usually higher than 80% and with a typical range from 78–85% according to Kerr et al. [29]. Glycerol is a 3-carbon alcohol, which is widely found in many animal tissues either as a free molecule or as the structural backbone of triacylglycerol molecules. At a metabolic level, glycerol can be converted to glucose via gluconeogenesis or it can be oxidized via glycolysis and incorporated into the tricarboxylic acid cycle to generate energy [30]. As a gluconeogenic precursor or a glycolytic substrate, glycerol provides energy that could benefit newly weaned piglets due to the higher energy requirement for maintenance [31] and, concurrently, the low feed intake immediately after weaning [32].

To date, there has been little research examining the effect of replacing lactose or lactose-rich ingredients with glycerin. We aimed to compare the effects of glycerin as it replaced sweet whey in pre-starter diets for weaned pigs. A high lactose content in the diet improves the growth performance of piglets as well as the growth of beneficial bacteria, in particular Lactobacillus, with the positive effects being more pronounced in the first two weeks after weaning [15].

From a productive point of view, our results showed that the ADG increased quadratically by increasing the dietary glycerin content compared with piglets fed the control diet while the ADFI decreased linearly in the pre-starter period. In any case, the FCR for glycerin-fed piglets improved by 9% with respect to piglets fed the G0 diet despite piglets fed G5 having a lower ADFI. Shields et al. [10], who evaluated glycerin inclusion up to 10% in the replacement of lactose in piglets weaned at 21 days, found that increased amounts of glycerin linearly increased the ADG and the ADFI but did not affect the gain to feed (G:F) ratio for the two first post-weaning weeks. By contrast, it is interesting to note that in another experiment conducted by the same authors, they found no difference in the growth performance of piglets with the inclusion of glycerin (5%) during the first feeding phase despite using the same source and batch of glycerin and conducting both studies using the same genetic line of pigs at the same facility. Nevertheless, in both experiments of this study, the lactose content of the control diet was 20% and the experimental diet with the highest level of glycerin (10%) still contained 10% lactose. In our study, the lactose levels were much lower, ranging from 6.2–2.7% for the G0 and G5 diets, respectively. On the other hand, in a recent study in which the basal diet containing lactose at intermediate levels (10%) and where weaned piglets were individually housed in metabolic cages and fed diets replacing the basal diet by 50, 100 or 150 g kg^−1^ of glycerin (or fed a diet in which the lactose was fully replaced by glycerin), no significant differences on the ADG, ADFI and FCR ratio were found for the first 12 post-weaning days [12]. 

Therefore, dietary glycerin levels in replacement of lactose produced different responses on the ADFI, ADG and FCR (or G:F) of piglets during the first feeding phase after weaning. The variability in these observations on the growth performance of glycerin-fed piglets may not only be associated with the levels of glycerin in the diets and their composition but also the basal level of lactose in the control diet as well as the potential interactions with other feed ingredients. In addition, weaning stress greatly influences the pig performance after weaning and could also be a major cause of variation in the response of pigs to different levels of dietary glycerin.

In the current study, the second feeding phase or starter period was initiated 24 days after weaning and lasted 15 days where crude glycerin was included as a replacement for wheat in starter diets. While the highest ADFI and worst FCR were found in piglets fed the G2.5 diet, there was no difference between the G5 and control (G0) diets. Taking into account the whole post-weaning period (pre-starter + starter), the dietary treatment did not affect the ADG whereas the increasing amounts of dietary glycerin either tended to decrease the ADFI or linearly decrease the FCR. 

Along these lines, other authors have also addressed the addition of glycerin to piglet post-weaning diets as a partial replacement of cereals. Zijlstra et al. [9] studied the addition of glycerin (at 0, 4 and 8%) to replace wheat in diets during a four week study with piglets of 7.3 kg live weight and selected at 27–30 days of age. Piglets fed 8% glycerin were 1.11 kg heavier than those fed 0% glycerin although this effect could be due to the high content of ether extract (EE) of the crude glycerin used (15.6%). In addition, glycerin inclusion tended to linearly increase the ADG and increase quadratically the ADFI without influencing feed efficiency. Similarly, the inclusion of 3 or 6% crude glycerin replacing corn in diets for 11–27 kg pigs increased the ADG as a result of an increased ADFI without affecting feed efficiency [8]. The degree of purity of the glycerin used in the latter experiment was similar to that of our study. On the other hand, Lammers et al. [3] reported that pigs (from an initial BW of 7.9 kg) could be fed corn-soybean meal-based diets and including crude glycerin up to 10% without the ADG, ADFI and G:F being affected in any feeding stage over a 138 day period. More recently, the inclusion of glycerin (up to 6%) in sorghum-soybean diets has also been evaluated, reporting a quadratic effect on growth performance traits (ADG and ADFI) in post-weaning piglets (23–70 days) [33].

When comparing our findings with those of previous studies, the degree of purity or content of glycerol and the origin and levels tested of crude glycerin, which can affect its composition and nutritional value, as well as the housing, feeding and management of piglets (commercial vs. experimental conditions), should be taken into account. Moreover, significant results could be also related to the precision of the estimates and the power of each study to detect statistical differences according to the sample size.

The current results showed that the ATTD coefficients of DM, OM and CP improved as glycerin increased in both feeding periods (pre-starter and starter) so the digestibility was higher in piglets fed diet G5. Consequently, the lower feed intake found in piglets fed the G5 diet could be probably compensated by a higher digestibility of this dietary treatment in comparison with other groups although these results should be interpreted with caution. 

Human and animal studies have indicated that glycerol is rapidly absorbed in the intestine [34] where absorption involves an Na(+)-dependent carrier-mediated transport system [35,36]. Nevertheless, glycerol could be also absorbed by the stomach (likely by passive diffusion) but at a rate of absorption slower than that in the intestine [34]. It should be noted that the absorption rate of glycerol is about one-fourth that of glucose where maximum serum levels in humans were observed 1–2 h after ingestion [37]. Moreover, a high ileal digestibility of glycerol (higher than 99%) has been previously reported in piglets [11]. Therefore, the digestibility of crude glycerin used in animal feed depends on its glycerol content [20]. 

In this line, the energy digestibility of crude glycerin with more than 80% glycerol ranged between 89 and 92% in piglets [38]. Verussa et al. [39] showed that there was an increase in the fecal digestibility of the DM and OM fractions as the crude glycerin inclusion increased up to 15% in a starter diet, which was in accordance with our digestibility results using a glycerin of a very similar composition (865 g/kg glycerol). Likewise, fecal digestibility coefficients of OM and EE were affected by dietary treatment, increasing linearly with increasing crude glycerin levels (up to 5%) in growing-finishing pig diets [27]. These findings may be explained by the fact that glycerin inclusion represents an indirect addition of organic matter also including EE and this might have led to a better use of these fractions. However, the inclusion of glycerin at 9 and 18% to piglet diets did not affect the ileal digestibility of DM and CP but increased the levels of urinary glycerol [11]. Similarly, no significant effect on the total tract apparent digestibility coefficients of DM, CP and gross energy (GE) were reported for piglets fed diets containing high levels of glycerin (maximum 15%) whereas urinary production and GE in urine increased with rising levels of glycerin [12]. Taking in mind that excess glycerol cannot be converted to glycerol-3-phosphate by glycerol kinase (GK) in the liver and that this surplus glycerol could be excreted in the urine [5], these results would suggest that the metabolic pathways of glycerol utilization could be saturated when high levels of glycerin are used. However, Papadomichelakis et al. [40] found that liver GK mRNA expression of 72 day old pigs increased linearly in response to increasing the crude glycerin inclusion (up to 15% diet) without finding any saturation effects of this enzyme.

Finally, the current results showed that there were also no differences between dietary treatments in terms of blood metabolites measured: insulin and total and acylated ghrelin. Insulin is a hormone whose levels usually increase after feeding to provide energy to the cells, decreasing blood glucose levels. However, no effect of crude glycerin on blood glucose or insulin concentration was observed in piglets [10] growing [41] or finishing pigs [3,42,43]. Ghrelin, in turn, is a fast-acting hormone that increases appetite so it is also known as the “hunger hormone”. It plays an important role in energy balance and circulates in two forms: acylated and unacylated ghrelin. Although acylated ghrelin is the primary bioactive form, levels of the total and acylated forms are closely associated with one another across a wide variety of physiological mechanisms that affect ghrelin concentrations [44,45]. In this regard, our findings showed a strong negative correlation between both ghrelin forms. Furthermore, ghrelin levels have been shown to be reciprocal to glucose and insulin levels, which may regulate ghrelin release [46] although there are also contrary reports indicating that plasma ghrelin concentrations are not regulated by glucose or insulin in healthy young humans [47]. In a previous work [42], it was found that there was a a tendency for higher acyl-ghrelin levels in Iberian crossbred pigs from 50 to 100 kg BW fed 10% glycerin, which were associated with a higher feed intake. In addition, pregnant sows fed diets containing glycerin (up to 6%) had lower concentrations of acyl-ghrelin and higher concentrations of leptin at 0 minutes after feeding [48] suggesting that the dietary supplementation of glycerin may have exerted a satiating effect. However, to our knowledge, the effect of glycerin inclusion on the serum concentration of ghrelin has never been previously described in piglets and requires further research.

## 5. Conclusions

The current findings showed that crude glycerin could be used up to 2.5% in piglet diets without worsening the growth performance during the first post-weaning weeks. Considering the whole post-weaning feeding period and when glycerin was included at 5%, the results showed that the FCR and ATTD coefficients were improved although the ADFI tended to be lower. Therefore, crude glycerin could be not only a partial substitute for lactose-rich ingredients but also as an alternative ingredient to replace cereals in post-weaning piglet diets.

## Figures and Tables

**Table 1:** Ingredients and composition of diets (as-fed basis).

Item	Pre-Starter ^1^			Starter ^1^		
	G0	G2.5	G5	G0	G2.5	G5
Ingredients, %						
Corn	31.82	31.36	31.18	35.00	35.00	35.00
Wheat	15.00	15.00	15.00	15.81	12.63	9.65
Soybean meal, 440 g CP/kg	11.55	12.00	12.00	23.92	24.72	25.50
Barley	10.00	10.00	10.00	15.00	15.00	15.00
Nutralmix 10% Primafeed SM premix ^2^	10.00	10.00	10.00	--	--	--
Actium FR ^3^	7.50	7.50	7.50	--	--	--
Sheep sweet whey powder	7.50	5.00	2.50	--	--	--
Soy oil	2.99	2.76	2.47	5.37	5.28	5.00
Whey powder, 50% fat	2.50	2.50	2.50	--	--	--
Crude glycerin >80% ^4^	0	2.50	5.00	0	2.50	5.00
Monocalcium phosphate	--	--	--	1.35	1.35	1.35
Actium Nursery Booster Premix ^5^	--	--	--	1.00	1.00	1.00
Calcium carbonate	--	--	--	0.76	0.76	0.75
Titanium dioxide	0.50	0.50	0.50	0.50	0.50	0.50
Sodium chloride	--	--	--	0.40	0.40	0.40
L-Lysine HCl	0.32	0.34	0.38	0.48	0.46	0.45
L-Threonine	0.15	0.35	0.77	0.20	0.19	0.19
DL-Methionine	0.13	0.14	0.15	0.18	0.18	0.18
L-Tryptophan	0.04	0.05	0.05	0.03	0.03	0.03
Calculated composition ^6^, %						
Net Energy, kcal/kg	2540	2540	2540	2525	2525	2525
Lysine digestible, %	1.28	1.28	1.28	1.13	1.13	1.13
Lactose, %	6.27	4.52	2.77	--	--	--
Analyzed composition ^7^, % on dry matter (DM) basis except for DM						
DM	91.2	90.9	91.0	89.6	89.5	89.5
Ash	5.40	5.14	5.37	6.12	6.23	6.17
Crude protein (CP)	18.3	18.9	18.7	18.4	18.3	18.7
Starch + sugars	42.5	42.6	42.4	44.7	44.0	43.0
Crude Fiber (CF)	3.64	3.78	3.55	4.21	4.25	4.36

^1^ Dietary treatments: 0, 2.5 and 5% crude glycerin included in G0, G2.5 and G5, respectively. ^2^ Nutralmix 10% Primafeed SM (Nutral, S.A., Madrid, Spain). Supplied per kg of complete diet: crude protein, 2.3 g; ether extract, 0.8 g; crude fiber, 0.35 g; ash, 1.95 g; vitamin A, 14,000 IU; vitamin D3, 2000; vitamin E (alfa-tocoferol), 60 mg; copper sulfate pentahydrate, 150 mg. ^3^ Actium FR (Nutral, S.A., Madrid, Spain). Composition: crude protein, 41.6%; ether extract, 6.1%; crude fiber, 4.4%; ash, 16.2%; lysine, 5%. ^4^ Chemical composition: glycerol, 85.00%; methanol, <0.5%; moisture, 9.22%; ash, 4.51%; phosphorus, 0.11%; sodium, 1.66% and potassium, 0.05%. ^5^ Actium Nursery Booster Premix (Nutral, S.A., Madrid, Spain). Supplied per kg of complete diet: vitamin A, 7500 IU; vitamin D3, 750; vitamin E (alfa-tocopherol), 45 mg; vitamin K3, 3.8 mg; vitamin B1, 1.5 mg; vitamin B2, 4 mg; vitamin B6, 3.8 mg; vitamin B12, 0.038 mg; niacin, 15 mg; calcium pantothenate, 11 mg; choline chloride, 225 mg; folic acid, 2.2 mg; biotin, 0.15 mg; zinc oxide, 75 mg; manganese oxide, 23 mg; ferrous sulphate heptahydrate, 121 mg; copper sulfate pentahydrate, 10 mg; sodium selenite, 0.22 mg; potassium iodide, 0.150 mg. ^6^ According to FEDNA (Fundación Española para el Desarrollo de la Nutrición Animal) [18]. ^7^ Based on analyses in duplicate per dietary treatment and corresponding to the average of each feeding period.

**Table 2:** The effect of adding glycerin to feed on body weight (BW), average daily gain (ADG), average daily feed intake (ADFI) and feed conversion ratio (FCR) of piglets in the pre-starter and starter feeding period.

Growth Performance	Diets ^1^			SEM ^2^	*p*-Value ^3^	
	G0	G2.5	G5		L	Q
Number of pens	6	6	6			
BW, kg						
Start of the study (at 0 day)	7.74	7.73	7.73	0.221	0.982	0.983
End of pre-starter period (at 24 day) ^4^	15.02 ^ab^	15.60 ^a^	14.77 ^b^	0.134	0.456	0.026
End of starter period (at 39 day) ^5^	22.24	21.79	21.74	0.140	0.168	0.537
Pre-starter period, 0–24 d						
ADG, kg day^−1^	0.298 ^ab^	0.324 ^a^	0.292 ^b^	0.006	0.647	0.034
ADFI, kg day^−1^	0.433 ^a^	0.426 ^ab^	0.397 ^b^	0.006	0.029	0.407
FCR, kg feed kg^−1^ gain	1.453 ^a^	1.317 ^b^	1.367 ^b^	0.014	0.021	0.006
Starter period, 24–39 day						
ADG, kg day^−1^	0.473	0.445	0.447	0.009	0.243	0.436
ADFI, kg day^−1^	0.772 ^ab^	0.804 ^a^	0.728 ^b^	0.012	0.166	0.056
FCR, kg feed kg^−1^ gain	1.634 ^a^	1.806 ^b^	1.638 ^a^	0.026	0.959	0.008
Whole period, 0–39 d						
ADG, kg day^−1^	0.365	0.370	0.351	0.005	0.275	0.267
ADFI, kg day^−1^	0.562 ^a^	0.571 ^a^	0.524 ^b^	0.008	0.060	0.113
FCR, kg feed kg^−1^ gain	1.540 ^a^	1.540 ^a^	1.492 ^b^	0.009	0.039	0.210

^1^ Dietary treatments: 0, 2.5 and 5% crude glycerin included in G0, G2.5 and G5, respectively. ^2^ SEM: standard error of the mean. ^3^ Linear (L) and quadratic (Q) contrasts. ^4^ BW adjusted for differences in initial weight being the regression coefficient for BW at 0 d (kg): 1.171 ± 0.156 (*p* < 0.001). ^5^ BW adjusted for differences in initial weight being the regression coefficient for BW at 24 d (kg): 1.038 ± 0.125 (*p* < 0.001). ^a,b^ Means within a row with different letters were significantly different at *p* ≤ 0.05.

**Table 3:** The effect of adding glycerin to pig feed on the apparent total tract digestibility (ATTD) of dry matter (DM), organic matter (OM) and crude protein (CP).

ATTD	Diets ^1^			SEM ^2^	*p*-Value ^3^	
	G0	G2.5	G5		L	Q
Number of pens	6	6	6			
Pre-starter period						
DM, %	80.3 ^a^	81.2 ^a^	82.4 ^b^	0.175	<0.001	0.684
OM, %	82.4 ^a^	83.3 ^a^	84.5 ^b^	0.169	<0.001	0.580
CP, %	74.6 ^a^	77.5 ^b^	80.6 ^c^	0.202	<0.001	0.730
Starter period						
DM, %	79.9 ^a^	78.5 ^a^	83.9 ^b^	0.571	0.012	0.013
OM, %	82.2 ^a^	80.8 ^a^	85.9 ^b^	0.495	0.008	0.008
CP, %	78.1 ^a^	74.4 ^b^	80.3 ^a^	0.679	0.196	0.004

^1^ Dietary treatments: 0, 2.5 and 5% crude glycerin included in G0, G2.5 and G5, respectively. ^2^ SEM: standard error of the mean. ^3^ Linear (L) and quadratic (Q) contrasts. ^a,b,c^ Means within a row with different letters were significantly different at *p* ≤ 0.05.

**Table 4:** The effect of glycerin addition on the serum concentration of total and acylated ghrelin and insulin. Data were logarithmically transformed using a log 10 scale; means (and standard errors of the mean) are presented in the original scale.

Serum Concentration	Diets ^1^			SEM ^2^	*p*-Value ^3^	
	G0	G2.5	G5		L	Q
Sample size ^4^	12	12	12			
Total ghrelin, pg/mL	592.9	615.2	537.0	47.238	0.606	0.599
Acylated ghrelin, pg/mL	114.0	51.9	93.8	30.924	0.711	0.152
Insulin, µg/mL	5.79	3.70	7.10	0.868	0.614	0.135

^1^ Dietary treatments: 0, 2.5 and 5% crude glycerin included in G0, G2.5 and G5, respectively. ^2^ SEM: standard error of the mean. ^3^ Linear (L) and quadratic (Q) contrasts. ^4^ Sample size: two animals per pen and six pens per treatment.

## Data Availability

Data sharing is not applicable to this article.

## Supplementary Materials

The following are available online at https://www.mdpi.com/article/10.3390/ani11051249/s1, Table S1: Pearson’s correlation matrix for serum concentrations of total ghrelin, acylated ghrelin and insulin at the end of the study (on day 39).
