# Supplementary material for: The Effect of the Dietary Inclusion of Crude Glycerin in Pre-Starter and Starter Diets for Piglets
Source: Animals (Basel). 2021 Apr 26;11(5):1249. doi: 10.3390/ani11051249 (PMC8145888; doi:10.3390/ani11051249)
Supplement: Supplementary file 1 [file animals-11-01249-s001.zip › animals-1160088-supplementary.pdf]

Table S1. Pearson's correlation matrix for serum concentrations of total ghrelin, acylated ghrelin, and insulin at the end of the study (on day 39). Data were logarithmically transformed using a log10 scale. Sample size (n) = 36.

	**Acylated ghrelin**	**Insulin**
Total ghrelin	-0.686 *	0.422
Acylated ghrelin		-0.489

Note: "*"indicate significant correlation at ≤ 0.05 level.
